# A synthetic data-driven machine learning approach for athlete performance attenuation prediction

**DOI:** 10.3389/fspor.2025.1607600

**Published:** 2025-05-27

**Authors:** Mauricio C. Cordeiro, Ciaran O. Cathain, Lorcan Daly, David T. Kelly, Thiago B. Rodrigues

**Affiliations:** ^1^ Department of Engineering & Informatics, Technological University of the Shannon, Athlone, Ireland; ^2^ Department of Sport & Health Sciences, Technological University of the Shannon, Athlone, Ireland; ^3^ SHE Research Centre, Technological University of the Shannon, Athlone, Ireland

**Keywords:** synthetic data, performance prediction, machine learning, tabular variational autoencoders, athlete monitoring

## Abstract

**Introduction:**

Athlete performance monitoring is effective for optimizing training strategies and preventing injuries. However, applying machine learning (ML) frameworks to this domain remains challenging due to data scarcity limitations. This study extends previous research by evaluating Tabular Variational Autoencoders (TVAE) for generating synthetic data to predict performance attenuation in Gaelic football athletes.

**Methods:**

This study assesses synthetic data quality through a comprehensive evaluation framework combining column shape similarity metrics and Hellinger distance analysis, quantifying distributional fidelity across individual variables. Our ML implementation follows a two-phase approach. In the first phase, we evaluated models trained on hybrid datasets with varying synthetic proportions (10%–100%). In the second phase, we examined models trained exclusively on synthetic data and tested them on real data to analyze the utility of the synthetic data.

**Results:**

Our results demonstrate that TVAE-generated synthetic data closely replicates original distribution patterns, achieving 85.53% column shape similarity and a Hellinger distance of 0.169. Models trained with additional synthetic data or exclusively on synthetic data outperformed real-data baselines across multiple metrics, particularly for neuromuscular parameters. Our findings emphasize that this approach increased data availability and improved model performance in specific scenarios.

**Discussion:**

Several limitations remain: (1) there is limited framework transferability to sports with different physiological demands; (2) the Synthetic Data Generation (SDG) does not currently enforce feature constraints, and future implementations must ensure the procedure respects domain-specific feature limits; and (3) TVAE faced data fidelity challenges with certain variables, such as VO_2max_. These findings demonstrate the utility of synthetic data for predicting performance attenuation in Gaelic Football athletes. They address the challenge of data scarcity and highlight how synthetic data can be effectively integrated across physiological, neuromuscular, and perceptual metrics in athlete monitoring. This opens new possibilities for exploring similar classification tasks in sports performance analysis.

## 1 Introduction

The interplay of fitness components, match-day performance, and recovery from match-play offers a significant opportunity for data-driven performance monitoring (1). In Gaelic football, athletes' performance involves a combination of high-intensity actions and aerobic demands, leading to fatigue and muscle damage influenced by physical, mental, and metabolic factors (1–3). Performance attenuation refers to a decline in physical and mental performance during or after demanding activities. It results from accumulated fatigue, muscle damage, and several physiological factors, including metabolite build-up, reduced muscle contractility, and depleted glycogen stores (1–4). Addressing this issue is essential, as it directly impacts an athlete's ability to maintain peak performance and recover adequately between matches. To decode these complex interactions, machine learning (ML) can reveal non-linear patterns in performance metrics datasets, enabling a predictive-based understanding of performance predictors (5, 6). However, creating high-quality datasets remains challenging because of privacy concerns and high costs associated with data collection, including qualified personnel and expensive monitoring systems (6). Therefore, synthetic data generation (SDG) offers a viable solution, augmenting datasets while preserving the statistical properties of real-world data, improving ML applicability in sports analytics (7).

SDG methods range from basic statistical techniques to advanced generative algorithms for generating synthetic tabular data. Statistical approaches such as masking, coarsening, and mimicking (8, 9) are easy to implement but struggle to preserve inter-column relationships. Joint distribution sampling (10) improves relationship preservation but faces scalability challenges with complex datasets. Thus, sophisticated algorithms are needed to capture individual patterns in sports performance data, which often includes multi-modal metrics such as neuromuscular, perceptual, and biochemical responses. However, each algorithm has its advantages and limitations, and choosing an appropriate method is a nuanced decision based on the available data, specific goals, and computational resources (11).

Recent advancements in deep learning have popularized generative algorithms for data synthesis, with Variational Autoencoders (VAEs) and Generative Adversarial Networks (GANs) emerging as leading approaches (12). While GANs excel in generating high-fidelity synthetic images, their performance on tabular data, particularly mixed datasets with continuous and categorical variables, has shown limitations in capturing full data diversity and maintaining training stability (13–15). Moreover, training and evaluating GANs is challenging due to their sensitivity to random initialization and hyperparameter settings, often causing generators with similar architectures to behave unpredictably (16). In light of these challenges, this study focuses on VAEs for SDG, specifically TVAE (Tabular Variational Autoencoder) (17), ensuring its ability to generate synthetic data replicating the original dataset's relationships and statistical properties (18–20).

SDG applications in sports science have advanced in recent years, with studies demonstrating their potential to address data scarcity limitations (21). used VAEs to generate synthetic posture data, effectively capturing biomechanical relationships and improving model training outcomes, though limitations in replicating high-precision details were noted in their analysis. Similarly (22), applied VAEs, generative adversarial networks (TimeGAN), and Autoregressive Denoising Diffusion Models (TimeGrad) to synthesize athlete time-series data (e.g., sleep quality, mood, training load), achieving superior fidelity when using TimeGAN but facing challenges in generalizing results because of their biological data complexity and small sample sizes (five athletes). These examples highlight the need for SDG methods that preserve complex inter-domain relationships and generate sufficient samples to overcome statistical limitations related to data scarcity.

Integrating ML and SDG can address challenges in sports analytics, such as improving dataset diversity for accurate performance attenuation predictions. By augmenting limited real-world datasets with synthetic samples generated via TVAEs, this approach can reduce overfitting risks and enhance model generalizability (7, 23) (e.g., for scenarios like atypical performance profile patterns that are underrepresented in small datasets). This study addresses three research questions: (1), Can TVAE-generated synthetic data effectively replicate the statistical properties of our athlete performance data? (2), To what extent does augmenting limited real data with synthetic samples improve performance attenuation prediction across physiological and perceptual metrics? (3), Can models trained exclusively on synthetic data perform as well as or better than those trained on real data for performance attenuation classification tasks?

To address these research questions and analyze the potential of synthetic data for performance attenuation prediction, we developed a comprehensive methodological framework. First, we evaluated the statistical fidelity of TVAE-generated synthetic data through multiple validation techniques, including Hellinger distance measurements, column shape analysis, and column pair trend assessments. We then established benchmark performance by training ML classifiers exclusively on real data. Following these preliminary steps, our framework employed a two-phase methodology combining ML models with TVAE-based SDG. The first phase evaluates predictive performance when training ML models on hybrid datasets containing real and synthetic samples at varying proportions. The second phase assesses the standalone utility of synthetic data by training models exclusively on TVAE-generated samples and testing them on real data, then evaluating them at different proportions of synthetic samples. This structured approach enables the investigation of optimal real-to-synthetic data ratios and explores whether synthetic data can serve as a viable substitute when access to real performance data is limited.

This approach diverges from previous research in sports analytics through SDG, which has predominantly employed traditional resampling techniques for class imbalance issues. Instead, we apply generative artificial intelligence through TVAEs to create high-dimensional synthetic performance data. While prior data augmentation investigations have demonstrated potential, whether ML models trained on synthetic data can perform comparably or superior to real-data baselines remains underexplored in sports performance literature. This exploration framework can determine the optimal synthetic data integration ratios for similar performance attenuation contexts and establish synthetic data's potential as a standalone training resource, extending beyond the class-balancing applications that have dominated previous sports analytics research.

Through this predictive modeling and synthetic data implementation approach, this work aims to advance the development of cost-effective, data-driven tools for performance monitoring in resource-constrained sports environments, where challenges such as limited data availability and computational resources are common.

## 2 Methods

### 2.1 Data source and participants

The data were obtained from the performance attenuation and timeline of the recovery study (1); this is a tertiary exploratory analysis of this existing data. The secondary exploratory investigation focused on traditional balancing techniques (SMOTE, ROSE, and ADASYN) to address class imbalance issues, and this tertiary investigation introduces a different approach through generative artificial intelligence via Tabular Variational Auto-Encoder (TVAE) to create high-dimensional synthetic performance data.

This study included 41 active and healthy male senior club-level Gaelic football players, aged 18–32, with experience in resistance training and Gaelic football (mean ± SD, age: 23.3 ± 4.2 years; height: 178.3 ± 7.91 cm; body mass: 80.64 ± 9.47 kg, sum of 7 skinfolds: 81.3 ± 28.0, percentage body fat: 14.3 ± 5.2). More details about their experience can be found in (1).

Neuromuscular, perceptual, and biochemical markers were measured at various time points: pre-match, half-time, post-match, and 24- and 48-h post-match. The neuromuscular-related parameters included Drop Jump (DJ), DJ Contact time (in seconds), Reactive Strength Index, and Countermovement Jump (CMJ) in centimeters (cm). Strength was assessed via one-repetition maximum (1RM) for Squat and Hip Thrust, measured in kilograms (kg). Regarding physiological parameters, Creatine Kinase (CK) levels were measured in international units per L (IU/L) and used to indicate an estimate of muscle damage. Anthropometric measurements included body mass (kg) and body fat percentage. Cardiorespiratory fitness was evaluated through VO_2max_ (in ml/kg/min). Additionally, Distance Total (meters), Total Accelerations, Total Sprints (>20 km/h), and Total Explosive Distance (meters) were implemented and captured using 18 Hz GPS units (24). Finally, a 5-question Likert Scale Questionnaire evaluated perceptual responses, assessing subjective aspects such as fatigue, sleep quality, muscle soreness, mood, and stress levels on a scale from 1 to 5, capturing athletes' self-reported well-being and performance-related perceptions (25).

Among these variables, the Machine Learning (ML) models' input and output variables were distinguished (Table 1). The input variables comprised all the above-cited anthropometric measurements, strength metrics, VO_2max_, total distance, sprints, accelerations, and baseline neuromuscular-related parameters. The temporal measurements of perceptual response, DJ, DJ Contact Time, RSI, CMJ, and CK levels were used to calculate athlete rankings through the methodology detailed in subsection 2.3, serving as the output variables for the models.

**Table 1:** The input and output variables.

Input	Output
^a^Baseline Drop Jump	Perceptual Response rank
^a^Baseline Drop Jump Contact Time	Creatine Kinase rank
^a^Baseline Countermovement Jump	Countermovement Jump rank
^a^Baseline Reactive Strength Index	Drop Jump rank
Age	Drop Jump Contact Time rank
VO_2max_	Reactive Strength Index rank
Body Mass	
Body Fat%	
1RM Hip Thrust	
1RM Back Squat	
Distance Total	
Total Accelerations	
Total Sprints	
Total Explosive Distance	

^a^ The input baseline values are not used in the output rankings calculation detailed in subsection 2.3, which shows that temporal measurements, e.g., pre/post-match values are used for that purpose.

### 2.2 Data preprocessing

**Figure 1:**
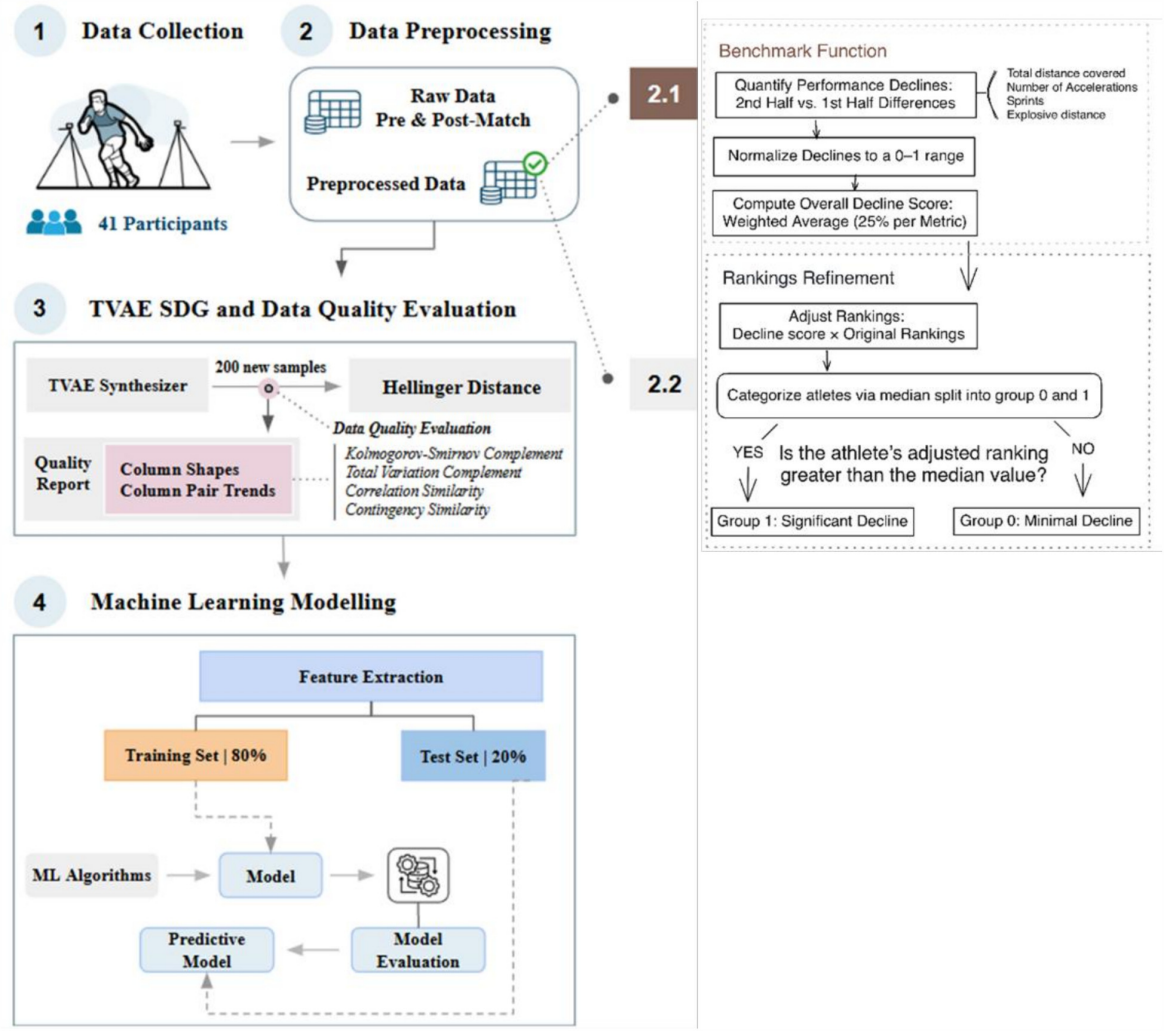
Flowchart of the methodology for predicting performance attenuation in gaelic football athletes. The steps consist of four systematic processes: (1) data collection of baseline and pre-/post-match metrics, (2) data preprocessing (benchmark function (2.1): intra-match decline calculation, normalization, weighted scoring; rankings refinement (2.2): adjusted rankings via median split; (3) Synthetic data generation using TVAE synthesizer and the data quality evaluation; (4) machine learning modeling. This framework addresses data scarcity while evaluating the quality of the synthetic data generated.

We implemented an athlete performance ranking system that prioritizes targeted variables, reduces data noise, and enables more accurate attenuation prediction by structuring data around key patterns (26), see Figure 1 for more details regarding this structure.

Following the rationale that physical abilities may be compromised during the latter stages of a match (27), we quantified performance attenuation through a ranking system based on pre-post-match differences. This tracking of differences between pre-match and post-match can provide relevant information about players' capability to cope with match demands. So, by using the Pandas library (28), we computed these differences for each output variable, e.g., a decrease from a pre-match CMJ height of 50 cm to a post-match height of 45 cm yielded a −5 cm difference.

To refine rankings, we introduced a benchmark function through a systematic process, which quantifies in-match performance attenuation by the differences computed between second-half and first-half values for four physical metrics: total distance covered, number of accelerations, sprints, and explosive distance. This methodological decision was based on findings related to the strength correlations between match running-related indicators and post-match muscle damage and neuromuscular performance declines (29, 30), while specifically designed to address inter-individual variability in our dataset. For example, consider an athlete whose distance covered drops from 1,000 m to 800 m (i.e., −200 m), with similar accelerations, sprints, and explosive distance declines. These raw declines are scaled to a 0–1 range (e.g., −200 m becomes 0.4) to normalize metrics with different units (e.g., meters, counts), ensuring fair comparisons. The normalized declines [e.g., (0.4, 0.5, 0.6, 0.3)] are averaged with equal weights (25% per metric) into an “overall decline score” (e.g., 0.45). This score is multiplied by pre-existing rankings to generate adjusted rankings, amplifying the intra-match performance attenuation impact. Finally, athletes are categorized into two subgroups: Group 0 (minimal decline, adjusted rankings ≤ median) and Group 1 (significant decline, adjusted rankings > median). For example, if the median adjusted ranking for CMJ is 0.5, athletes scoring ≤ 0.5 are assigned to Group 0, while those >0.5 are assigned to Group 1.

As mentioned, by encoding baseline performance and in-match performance decline, these rankings provide ML models with richer individualized characteristics, expecting an improvement in their ability to generalize across athletes with diverse attenuation patterns.

### 2.3 Synthetic data generation

#### 2.3.1 Tabular variational autoencoder (TVAE)

The Tabular Variational Auto-Encoder (TVAE) (17) is a deep generative model designed to synthesize realistic tabular data by adapting the Variational Auto-Encoder (VAE) framework (31). As noted in the introduction, TVAE was selected over alternative models due to its capabilities with tabular data containing continuous and categorical variables, addressing challenges when modeling complex athlete performance metrics (18–20). VAEs consist of an encoder-decoder architecture where the encoder maps input data into a low-dimensional latent space, and the decoder reconstructs the original data from these latent representations. In VAE, a regularization term is added over the latent space of the auto-encoder by adding a loss function to avoid overfitting (32). TVAE extends this framework, optimizing the Evidence Lower Bound (ELBO) loss function, which balances reconstruction accuracy and latent space regularization. The model employs the Adam optimizer with a learning rate of “1e-3” to refine synthetic samples. Further, the created synthetic data, *A* (*x*), can be kept as in Equation 1.

(1) $$\begin{array}{c} A\;(x)=\;B\;(\mathrm{Decomp}\;(\mathrm{Comp}\;(x)))\; \end{array}$$

Where x represents the actual performance dataset, B is the TVAE method with x as the input value and generates A (x). The Comp method, which acts as an encoder, masters the latent diffusions on actual data. Further, the Decomp method (Decoder) generates synthetic data by inspecting the latent diffusions. This methodology is supported in (33) and improved performance on real tabular datasets (17).

In our experiment, we implemented the TVAE using the SDV library (version 1.17.4) (34). TVAE inputs and outputs are shown in Table 1. Table 2 contains a summary of the configuration parameters used in this experiment. This configuration represents the model architecture and training specifications applied to the athlete performance dataset. This architectural configuration was selected following iterative evaluation of multiple parameter combinations, where each configuration was assessed using the *SDMetrics* library quality report measuring statistical fidelity across univariate distributions (column shapes) and multivariate relationships (column pair trends). The selected configuration demonstrated superior preservation of distributional characteristics.

**Table 2:** Configuration parameters implemented for the TVAE synthesizer.

Tool	Synthesizer	Configuration parameters
SDV (32)	TVAE (17)	Enforce_min_max_values: True
		Enforce_rounding: True
		Embedding_dim: 128
		Compress_dims: (128, 128)
		Decompress_dims: (128, 128)
		l2scale: 0.001
		Batch_size: 500
		Epochs: 500
		Loss_factor: 2

### 2.4 Synthetic data generation (SDG) quality assessment

#### 2.4.1 Synthetic data quality evaluation

To quantify distributional similarity between original and synthetic datasets, we employed the Hellinger distance analysis (35, 36). This statistical measure quantifies distributional similarity by directly comparing probability densities, with two distinct advantages for synthetic data evaluation. First, its bounded range [0–1] provides interpretational clarity, where distance approaching zero indicates near-identical distributions, and the distance of one indicates disjoint distributions. Second, it maintains dimensional consistency when aggregated across multiple variables, enabling systematic quality assessment across the entire feature space. These properties distinguish it from alternative metrics such as Kullback-Leibler divergence, which measures relative entropy, or Wasserstein distance, which quantifies the amount of distribution weight that must be moved and how far (37). The Hellinger distance is defined as Equation 2:

(2) $$\begin{array}{c} H(x,x^{\prime }=\frac{1}{\sqrt{2}}\sqrt{\underset{i}{\sum }{\left(\sqrt{q_{i}}-\sqrt{\;p_{i}}\right)}^{2}} \end{array}$$

Where $q_{i}$ and $p_{i}$ are the probabilities of every distinct result in *x* and *x^'^* variable spaces, respectively.

To complement the Hellinger distance analysis, we used the *Single Table Quality Report* from the *SDMetrics* library (38). This report evaluates the similarity between the real and synthetic datasets using two approaches: column shapes and column pair trends. “*Column Shapes*” measures the similarity between the real and synthetic datasets' marginal distributions (distributions of individual columns). The *Kolmogorov–Smirnov (KS) Complement* metric (39) is used for numerical and/or time-based columns, while the *Total Variation (TV) Complement* metric (40) is used for boolean and/or categorical columns. Moreover, “*Column Pair Trends*” measures the similarity between the relationships or trends between pairs of columns in real and synthetic datasets. The Correlation Similarity metric is used for pairs of numerical or time-based columns, the Contingency Similarity metric (41) is used for pairs of categorical or Boolean columns, and a combination of discretization and Contingency Similarity is used for pairs of numerical or time-based and categorical or Boolean columns.

Thus, by covering marginal and joint distributions, this report identifies areas where the synthetic data presents issues with features compared to the real data.

#### 2.4.2 Machine learning

To assess synthetic data utility in performance attenuation prediction, we employed four classification algorithms: Random Forrest (42), AdaBoost (43), XGBoost (44), and Linear Support Vector Machine (45). Our models' performance was assessed via precision, indicating the proportion of true positive predictions out of all positive predictions made by the model, the f1-score, providing the balancing of precision and recall of the model, making it helpful in evaluating performance in classification tasks where false positives and false negatives are important, and recall, measuring the proportion of true positive predictions out of all actual positives (46).

For all experimental conditions, including models trained on real data only and those incorporating synthetic data, we implemented a hyperparameter optimization protocol using grid search with stratified 5-fold cross-validation (47). For Random Forest, we optimized the number of estimators (50, 100, 200) and maximum tree depth (None, 10, 20, 30). For XGBoost, we tuned the number of estimators (50, 100, 200), learning rate (0.01, 0.1, 0.2), and maximum depth (3, 6, 9). AdaBoost optimization included the number of estimators (50, 100, 200) and learning rate (0.01, 0.1, 1.0), using decision tree classifiers with a maximum depth of 1 as base estimators. For Linear SVM, we optimized the regularization parameter C (0.01, 0.1, 1.0, 10.0) and maximum iterations (1,000, 2,000).

We implemented a two-phase experimental framework using 200 TVAE-generated synthetic samples for our experimental framework. It is important to note that our original dataset was already balanced by design through our median-split methodology mentioned in Section 2.2. This approach ensured near-equal-sized groups in the original data. We validated the preservation of this balanced distribution using synthetic data evaluation metrics from the SDMetrics package support (TVComplement score of 0.91 for categorical variables confirmed that our synthetic data preserved the balanced distribution present in the original dataset. Complementary to this, the KSComplement score of 0.92 for numerical variables demonstrated that we maintained the statistical properties of the performance metrics within each group and preserved the original balanced design.

Phase 1 — Hybrid Data Integration. In this phase, we evaluated the models' performance through additional synthetic data augmentation. The real dataset was divided into 75% training and 25% testing sets. The training set was augmented with synthetic samples at proportions ranging from 10% to 100%. Models were evaluated on the held-out real data test set to assess how synthetic augmentation affects generalization.

Phase 2 — Pure Synthetic Training. This phase tested whether models trained exclusively on synthetic data could predict performance attenuation in real athletes. Models were trained solely on synthetic samples at varying proportions (40%-100%) and evaluated against the complete real dataset. We limited our investigation to synthetic data proportions between 40% and 100% to ensure a sufficient sample size for reliable model training. At lower proportions, models exhibited high variance across validation folds, indicating instability in the learned patterns. The 40% threshold represents the empirically derived minimum proportion necessary to achieve stable model convergence while enabling a strong assessment of synthetic data's utility across a considerable range of proportions.

Results from both phases were benchmarked against all the best models' results for each ranking metric prediction when they were trained exclusively on real data using identical validation procedures. Therefore, in other words, only one model (i.e., the best model result) was selected for each performance metric benchmark comparison. This procedure enabled the assessment of synthetic data's utility while identifying optimal synthetic proportions for each performance metric and classification algorithm.

### 2.5 Statistical considerations

As described in [1], the original dataset was verified for normality using the Shapiro–Wilk test, and all variables met this criterion successfully (*p* > 0.05), ensuring a statistically sound basis for our subsequent analyses. The performance attenuation rankings were based on pre-post match differences statistically validated in the original study [1] via the multiple repeated measures ANOVAs with Bonferroni *post-hoc* analysis. These tests identified significant temporal changes in the key performance metrics (CK, PR, DJ, RSI, DJ Contact Time, and CMJ), which we subsequently used as ranking variables.

To deal with the possible issues of having a small sample size (*n* = 41) in our machine learning approach, we implemented methodological strategies as explained in Section 2.2. First, we employed a median-split methodology to create our ranking system, ensuring balanced representation between athletes who were experiencing minimal performance decline (Group 0) vs. significant performance decline (Group 1). This approach mitigated potential imbalance issues that could disproportionately affect model training with limited samples, especially when training the models using only real data.

While we acknowledge that the small sample size constrains the generalizability of our findings, these combined strategies allowed us to conduct a viable exploration of synthetic data's potential for performance attenuation prediction using this dataset's characteristics.

## 3 Results

**Figure 2:**
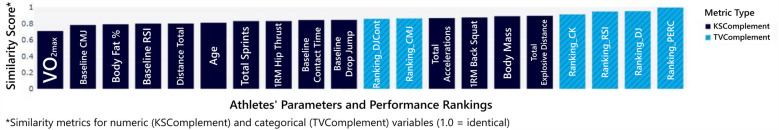
Illustration of the similarity between the real and synthetic datasets’ marginal distributions (individual column distributions), *n* = 41 and *n* = 200, respectively. The KSComplement, used in numerical columns, measures the maximum difference between cumulative distribution functions, and the TVComplement, used in categorical columns, quantifies the absolute difference in probability distributions. Higher scores indicate stronger alignment between the real and synthetic data, with a maximum score of 1.0 representing identical distributions.

**Figure 3:**
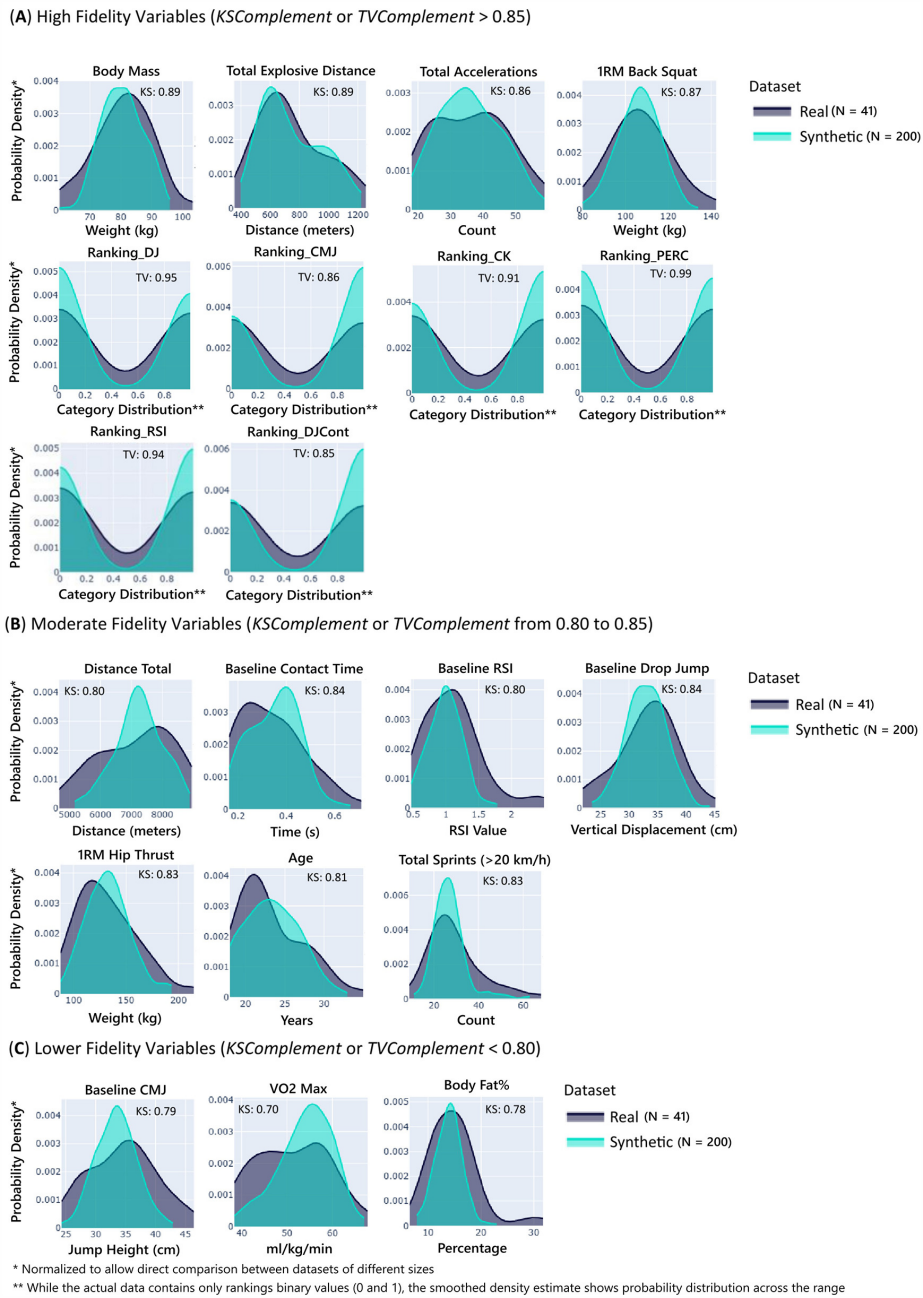
Distribution comparison between original and synthetic data with fidelity metrics. Variables are stratified into three categories based on their distributional similarity scores: **(A)** high fidelity (similarity > 0.85), **(B)** moderate fidelity (similarity 0.80-0.85), and **(C)** lower fidelity (similarity < 0.80). In each subplot, the green curve represents the distribution of a variable in the synthetic dataset, while the gray curve shows the corresponding distribution in the original dataset. Each distribution includes its specific *KSComplement* or *TVComplement* score, quantifying the degree of distributional alignment. This hierarchical visualization framework demonstrates variable-specific synthetic data quality, with categorical variables achieving strong replication fidelity (0.92), while variables such as VO_2max_ (0.70) present greater synthesis challenges.

The SDMetrics quality report evaluated the data fidelity across two approaches. The column shapes score of 85.53% demonstrated strong performance in replicating individual variable distributions, where the scores of KSComplement and TVComplement remained above 0.7, confirming that the synthesizer accurately captured the statistical properties of features (see Figure 2). Meanwhile, the column pair trends score of 79.97% reflected moderate success in preserving relationships between variables. The overall score of 82.75%, calculated as the average of these two components, indicates that the synthetic data reproduces approximately 83% of the original dataset's statistical patterns on a scale where 100% represents perfect replication. These quantitative results align with the visual assessment in Figure 3, where the green curves, representing the synthetic data distributions, align well with the gray curves, representing the original data distributions.

Moreover, the Hellinger distance of 0.168 demonstrated strong distributional similarity between synthetic and original datasets, with this metric's bounded range [0–1] providing interpretational clarity (where 0 indicates identical distributions). Variable-specific analysis revealed a pattern of synthesis fidelity: performance metrics such as Total Explosive Distance (0.064) and Total Accelerations (0.079) exhibited exceptional distributional alignment, while other parameters, including %Body Fat (0.237) and Baseline CMJ (0.247), presented relatively greater synthesis challenges. These Hellinger distances corroborate the quality report's overall score, showing the reliability of the synthetic dataset for applications requiring fidelity to the original data's statistical properties while identifying opportunities for refinement.

Table 3 shows the best ranking predictions for different models using only real data (as mentioned in the methods section, it is referred to as the benchmark). AdaBoost and XGBoost provided the best results for most metrics, with variations in performance across different ranking classifications. The creatine kinase metric achieves the highest performance, with XGBoost yielding an accuracy of 0.72, an F1 score of 0.69, and precision and recall values of 0.83 and 0.70, respectively. In contrast, the DJ metric shows the lowest performance, with XGBoost achieving an accuracy, F1 score, precision, and recall close to 0.45. These results are important for our comparison with the addition of synthetic data in the models' training (phase 1) and models trained exclusively with synthetic data (phase 2).

**Table 3:** Best ranking prediction model results using only real data.

Metric rank	Model	Best parameters	Accuracy	F1 score	Precision	Recall
^b^PR	Adaboost	^a^LR: 0.01, NE: 50	0.54	0.48	0.52	0.53
^b^CK	XGBoost	^a^LR: 0.01, MD: 3, NE: 50	0.72	0.69	0.83	0.70
^b^CMJ	XGBoost	^a^LR: 0.2 MD:3 NE: 50	0.45	0.45	0.45	0.45
^b^DJ	XGBoost	^a^LR: 0.01 MD: 3 NE: 50	0.45	0.41	0.42	0.43
^b^DJCT	AdaBoost	^a^LR: 0.1, NE: 50	0.63	0.61	0.65	0.62
^b^RSI	AdaBoost	^a^LR: 0.01, NE: 200	0.54	0.55	0.55	0.55

^a^ MD, maximum depth; MSS, minimum Samples_Split; NE, number of estimators; C, regularization parameter; LR, learning rate, MI, max iteration.

^b^ PR refers to perceptual response, encompassing subjective measures of athlete well-being and perceived exertion. CK denotes creatine kinase, a biochemical marker used to assess the extent of exercise-induced muscle damage. CMJ stands for countermovement jump, a common test for evaluating lower-body explosive power. DJ represents the drop jump, a plyometric exercise used to measure reactive strength and the effectiveness of the stretch-shortening cycle. DJCT is the drop jump contact time, referring to the ground contact duration during a DJ; shorter times are generally associated with better neuromuscular efficiency and elastic reactive strength. RSI is the reactive strength index, calculated as jump height divided by ground contact time during a DJ; it quantifies an athlete's ability to rapidly transition from eccentric to concentric muscle action (i.e., reactive strength).

Table 4 shows the models for best-ranking metrics prediction and their ideal additional synthetic data achieved. The optimal combination of model, synthetic data ratio, and hyperparameters depends on the unique demands of each performance metric. AdaBoost and Random Forest dominate scenarios requiring moderate to high synthetic data, while XGBoost excels in low-data regimes. Specifically, Synthetic data ratios shaped classification outcomes: low (10%–20%) improves generalization for CMJ and RSI, moderate (50%) boosts reliability for PR, CK, and DJ, and high (70%) captures better patterns in DJCT.

**Table 4:** Best ranking prediction model results and its ideal additional percentage of synthetic data.

Metric rank	Model	Synthetic data %	Best parameters	Accuracy	F1 score	Precision	Recall
^b^PR	^a^AB	50%	^a^MD: 2 NE: 100	0.81	0.80	0.88	0.80
^b^CK	^a^RF	50%	^a^MD: none NE: 200	0.72	0.72	0.81	0.75
^b^CMJ	^a^XB	10%	^a^LR: 0.1 NE: 100	0.81	0.82	0.82	0.82
^b^DJ	^a^RF	50%	^a^MD: none NE: 50	0.81	0.80	0.88	0.80
^b^DJCT	^a^AB	70%	^a^MD: 3 NE: 200	0.81	0.80	0.88	0.80
^b^RSI	^a^AB	20%	^a^MD: 2 NE: 50	0.72	0.72	0.73	0.72

^a^ MD, maximum depth; MSS, minimum samples split; NE, number of estimators; C, regularization parameter; LR, learning rate; MI, max iterations; RF, random forrest; XB, XGBoost; AB, AdaBoost; NB, Linear SVM.

^b^ PR refers to perceptual response, encompassing subjective measures of athlete well-being and perceived exertion. CK denotes creatine kinase, a biochemical marker used to assess the extent of exercise-induced muscle damage. CMJ stands for countermovement jump, a common test for evaluating lower-body explosive power. DJ represents the drop jump, a plyometric exercise used to measure reactive strength and the effectiveness of the stretch-shortening cycle. DJCT is the drop jump contact time, referring to the ground contact duration during a DJ; shorter times are generally associated with better neuromuscular efficiency and elastic reactive strength. RSI is the reactive strength index, calculated as jump height divided by ground contact time during a DJ; it quantifies an athlete's ability to rapidly transition from eccentric to concentric muscle action (i.e., reactive strength).

.

**Figure 4:**
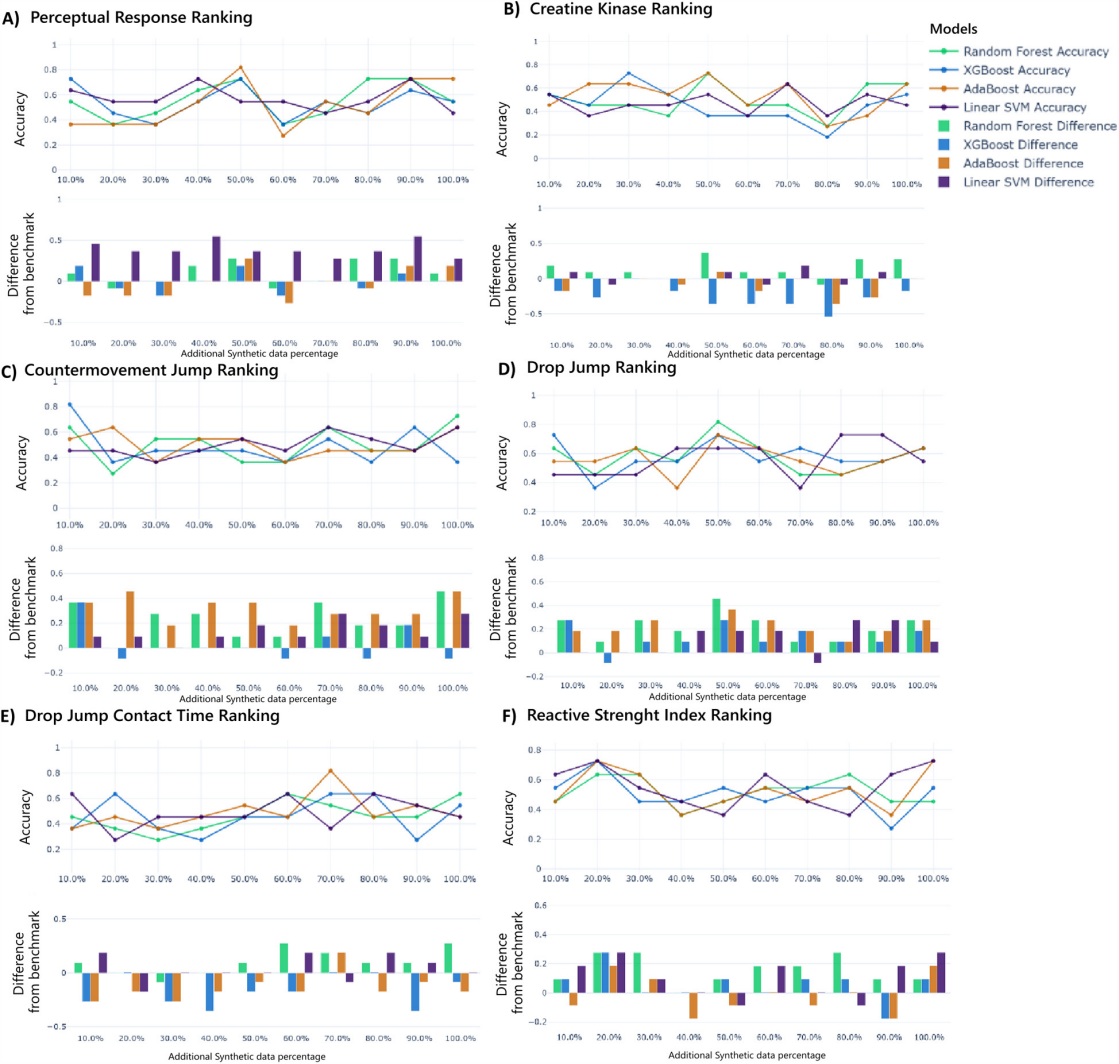
Ranking prediction models' performance with additional synthetic data for perception responses **(A)**, creatine kinase **(B)**, countermovement jump **(C)**, drop jump **(D)**, drop jump contact time **(E)**, and reactive strength index **(F)**. The upper graph shows the accuracy of each model over different additional synthetic data percentages, while the lower graph shows the difference in accuracy compared to the benchmark.

These metric-specific synthetic data ratios directly align with the performance trends in Figures 4A–F, where accuracy peaks align with their respective optimal synthetic data ratios (PR: 50%, CK: 50%, CMJ: 10%, DJ: 50%, DJCT: 70%, RSI: 20%), though some subplots reveal secondary peaks at alternative ratios. Further, benchmark comparisons quantify performance gains with more emphasis on PR, CMJ, DJ, and RSI rankings classification. This hybrid approach yielded improvements over real-data baselines, with accuracy gains of up to 50% for PR, CMJ, and DJ classifications when optimal synthetic proportions and model selection were applied. Moreover, to determine the size of these improvements, we computed standardized percentage effects at optimal proportions for all performance measures. The hybrid approach yielded effect sizes of 50.0% for PR, 0% for CK, 80.0% for CMJ, 80.0% for DJ, 28.6% for DJCT, and 33.3% for RSI, showing great significance for the majority of performance measures.

**Figure 5:**
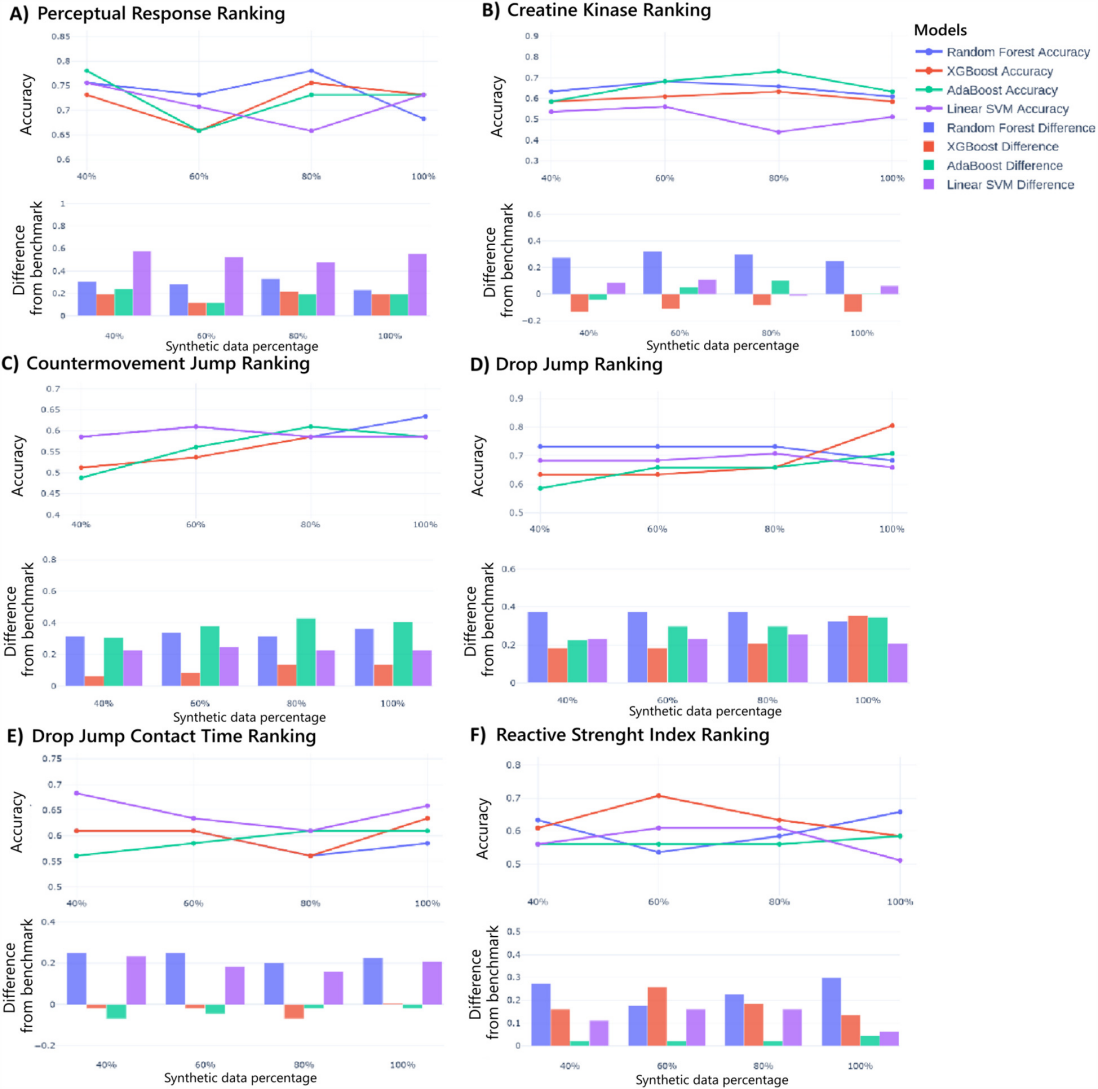
Models performance using only synthetic data for Perception Responses ranking prediction **(A)**, creatine kinase **(B)**, countermovement jump **(C)**, drop jump **(D)**, drop jump contact time **(E)**, and reactive strength index **(F)**. The upper graph illustrates the accuracy of each model across different synthetic data proportions, while the lower graph shows the difference in accuracy compared to the benchmark.

Moreover, after this analysis of hybrid real-synthetic data ratios uncovering optimization strategies specific to each ranking classification, Figures 5A–F explores data utility using models trained solely on synthetic data and testing them on real data. For PR and CK, accuracy improves steadily with higher synthetic data ratios. Most models surpass real-data baselines for PR, while only Random Forest clearly outperforms for CK. For CMJ and DJ, accuracy trends are consistent with increased synthetic data, and all models exceed real-data benchmarks. Moreover, minimal accuracy gains occur with increased synthetic data for DJ contact time, and only Random Forrest and Linear SVM models outperform real-data baselines. Finally, for RSI, accuracy remains stable across synthetic ratios, with most models outperforming real-data benchmarks. Moreover, the standardized effect sizes for models trained exclusively on synthetic data (at optimal proportions) were 33.3% for PR, 12.5% for CK, 60.0% for CMJ, 60.0% for DJ, 14.3% for DJCT, and 16.7% for RSI. These findings reveal that models trained exclusively on synthetic data can achieve comparable or superior performance to real-data-trained models across multiple metrics, particularly for neuromuscular-related parameters, demonstrating the potential of synthetic data as a standalone training resource for performance attenuation prediction.

Finally, performance variation across synthetic data percentages exhibited various model-specific patterns. Consequently, Linear SVM demonstrated greater sensitivity to synthetic data proportions, which can be attributed to its dependence on a singular optimized hyperplane defined by support vectors proximal to class margins, increasing its vulnerability to synthetic data addition. Therefore, these findings highlight the importance of model selection when employing synthetic data in similar applications applied to ML.

## 4 Discussion

This study investigates using SDG to improve performance attenuation prediction in Gaelic football athletes. Table 4, Figure 4,5 show Machine Learning (ML) models' effectiveness in predicting performance attenuation using phase 1 (hybrid-additional synthetic data) or phase 2 (only synthetic data).

The TVAE model effectively replicated the real data structure, confirmed by low Hellinger distance scores and aligning with previous studies that have demonstrated the capability of TVAEs to generate realistic synthetic data in tabular and high-dimensional data (18, 19). However, challenges with variables such as VO_2max_ suggest the need for TVAE retuning when working with small samples, as variational autoencoders can struggle with representation learning, posterior collapse, and model flexibility in such contexts (48–50).

The data scarcity issue is effectively mitigated through TVAE in this study. While balancing techniques remain important, Synthetic data generation (SDG) provided by generative models offers the potential to explore small datasets with greater flexibility, providing high-fidelity synthetic data that can unlock previously unexplored outcomes. By increasing the quantity and diversity of training data, and preserving the original distribution patterns, synthetic augmentation helps machine learning classifiers learn more robust feature relationships. This data procedure improved, in this case, the ML models' ability to detect true performance attenuation profiles that could be hidden in the limited original dataset. To achieve similar gains, researchers must guarantee that the synthetic data are of high quality and report clearly how they were created and how they were supposed to be applied, thereby increasing data availability and covering existing gaps in analysis.

Furthermore, while this study demonstrates the potential of synthetic data augmentation for improving classification performance, our model-centric approach presents methodological limitations in addressing domain-specific constraints and it is important to acknowledge that our findings are specific to Gaelic football and do not generalize to sports with different physiological demands and performance profiles, such as endurance sports. Future advancements in this SDG investigation could benefit from integrating data-centric AI frameworks (51) that dynamically profile datasets to guide model selection, enforce domain-specific constraints, and optimize synthetic data for downstream tasks, ensuring statistical fidelity and data utility. Such frameworks would streamline the synthesis process and ensure that generated data aligns with the requirements of complex real-world applications, particularly those involving temporal dependencies or heterogeneous populations. For example, parameters such as VO_2max_ exhibited the lowest synthesis fidelity. Therefore, a data-centric approach would first characterize these complex distribution patterns and variable interdependencies, then enforce domain-specific constraints (e.g., physiologically valid ranges and correlations) during the synthesis process. This constraint enforcement is important for sports performance applications where biological plausibility must be maintained. By embedding these constraints into the synthesis process, such frameworks would enhance the practical utility of predictive models in data-limited scenarios, facilitating more reliable, scalable, and domain-compliant SDG solutions (52).

In clinical and sports performance contexts, synthetic data generation approaches similar to those explored in our study could potentially address data analytics challenges in return-to-play assessment protocols. This represents an analogous application area where practitioners frequently encounter data limitations in the form of sparse longitudinal performance benchmarks when athletes resume competition following injury or extended absence. For example, sports scientists could implement our or a similar TVAE-based approach within an intelligent monitoring system that generates synthetic performance profiles based on limited historical data, enabling more reliable prediction of performance attenuation risks for athletes returning from injury. This system could alert coaches to potential declines in neuromuscular function before they manifest in competitive settings, allowing for timely training modifications.

This study, alongside studies such as (53), demonstrates the expanding scope of SDG applications within sports science. Our TVAE-based approach focuses on a specific use case, augmenting a limited dataset to enhance predictive modeling for performance attenuation monitoring (53). explored SDG using an alternative method, such as sequential tree-based algorithms, applied to a similar performance monitoring context. Specifically, they used a dataset from professional football players (*n* = 34) previously employed to investigate training load and injury relationships, adapting SDG for different purposes such as facilitating data sharing, reproducibility, exploration, and developing an education primer for potential application of these methods. These studies illustrate how SDG is adapted for diverse objectives, moving beyond simple data replication. Despite the different methodologies and target applications, both highlight that the expansion of SDG into any new use case requires attention to the generation process alignment with the target analysis and open documentation, as noted by (53).

### 4.1 Limitations

While we have mentioned slight limitations throughout this discussion section, it is important to highlight this study's constraints for better clarity.

First, the generalizability of our findings is constrained to the specific context of Gaelic football. Our results do not extend to sports with different physiological demands and performance profiles, such as endurance sports. The high-intensity demands of Gaelic football create unique performance attenuation patterns that are hard to manifest similarly in other sports contexts, limiting the transferability of our approach. Additionally, our sample consisted of male senior-level athletes, potentially limiting the applicability of our findings to female athletes or other level populations whose physiological responses to training and competition differ.

Moreover, our approach focused solely on the TVAE synthesizer. Future research should explore combining it with architectures such as TimeGAN and Diffusion Models to provide a broader assessment of these tools' potential in enhancing athlete monitoring. Although our TVAE implementation generated high-quality data, alternative models may be more effective at maintaining high fidelity in the generation of specific performance metrics, especially in addressing the challenges this study faced in preserving data fidelity for VO_2max_.

Our validation focused on statistical properties and predictive performance, rather than incorporating biological plausibility into the SDG process. This limitation emphasizes the importance of data-centric AI frameworks that enforce physiological validity during synthesis. Thus, future implementations should integrate constraint-based mechanisms to ensure biologically plausible conditions, particularly when generating synthetic data for rare or underrepresented performance profiles.

Finally, regarding ML classification tasks for similar applications, an important limitation to acknowledge is the binary-based classification of our current approach. Future investigations should incorporate, where feasible, multi-label classification rather than binary categorization, especially in groups where multiple performance attenuation patterns are expected. This extension would enable more granular profiling of performance decline responses and potentially improve predictive accuracy for athletes showing complex profiles.

## 5 Conclusion

This study employed TVAE-based SDG (synthetic data generation) to enhance performance attenuation prediction in Gaelic football athletes. Answering our first research question, TVAE effectively captured the overall statistical structure of the dataset (85.53% similarity), with specific parameters demonstrating lower replication fidelity, such as VO_2max_, %Body Fat, and Baseline CMJ. These replication challenges likely stem from our TVAE architecture variable-specific sensitivity and the limited sample size (*n* = 41). Answering our second and third research questions, our two-phase model performance analysis showed principal findings regarding data utility: first, the hybrid approach combining real and synthetic data improved classification performance when applying metric-specific optimal synthetic data proportion: PR classification accuracy increased by 50% (from 0.54 to 0.81) with 50% synthetic proportion, while CMJ and DJ classifications both achieved 80% improvements (from 0.45 to 0.81) with 10% and 50% synthetic proportions respectively. DJCT and RSI classifications showed more modest gains (28.6% and 33.3%) with 70% and 20% synthetic data respectively, while CK classification maintained consistent performance (0.72) with 50% synthetic data. Second, models trained exclusively on synthetic data frequently outperformed real-data baselines across multiple metrics, particularly for neuromuscular parameters. This finding extends beyond mere data augmentation to suggest synthetic data's potential as a viable alternative for primary model training resources in similar performance analytics implementations. In summary, phase one served an investigative function by identifying optimal mixing “synthetic and real data” ratios, and phase two addressed the practical question of synthetic data utility as a standalone resource. These findings demonstrated the potential of TVAE-generated synthetic data to improve performance attenuation prediction in Gaelic Football or similar sports demands, suggesting that synthetic data potentially addresses data scarcity challenges in similar sports performance monitoring dataset, where data availability constraints are common. Future studies should explore integrating multiple generative models and SDG domain-specific constraint enforcement to further enhance the fidelity and applicability of synthetic data solutions in athlete monitoring.

## Data Availability

The data analyzed in this study is subject to the following licenses/restrictions: the original data were collected by Lorcan Daly and are not publicly available due to ethical restrictions. Researchers may request access by contacting Lorcan Daly at lorcan.daly@tus.ie. The synthetic data generated during this study are publicly available at: https://github.com/mauriciomau0/Synthetic-Data-TVAE-for-Athlete-Performance-Attenuation-Prediction. Requests to access these datasets should be directed to Mauricio Cordeiro, mauricio.cordeiro@tus.ie.
